# Dataset of bovine mammary gland dry secretion proteome from the end of lactation through day 21 of the dry period

**DOI:** 10.1016/j.dib.2020.105954

**Published:** 2020-06-30

**Authors:** Timothy A. Reinhardt, John D. Lippolis

**Affiliations:** National Animal Disease Center, USDA/ARS, Ames, IA 50010, United States

**Keywords:** Dairy cow dry period, Bovine dry secretions, Proteome, Mastitis, Mammary involution

## Abstract

This dataset is a label-free quantitation of proteins milk and dry secretions from the end of lactation through day 21 of the dry period using liquid chromatography with tandem mass spectrometry (LC-MS/MS). The data supplied in this article supports the accompanying publication entitled “Characterization of bovine mammary gland dry secretions and their proteome from the end of lactation through day 21 of the dry period” [1]. The Thermo mass spectrometry raw files and MaxQuant files have been deposited to the ProteomeXchange Consortium via the PRIDE partner repository with the dataset number PXD017837.

**Specifications Table**

**Table:** 

**Subject**	Animal Science and Zoology
**Specific subject area**	Dairy Science. Specifically, the proteomic changes that occur in dry secretions during the dry period.
**Type of data**	Raw MS/MS data files and MaxQuant output files.
**How data were acquired**	LC-MS/MS (Thermo Orbitrap Velos Pro).
**Data format**	Thermo proprietary raw files and MaxQuant output files.
**Parameters for data collection**	Eleven cows were sampled on days 1, 3, 10 and 21 days of the dry period.
**Description of data collection**	Single quarter dry cow secretion samples were collected aseptically, from the same quarter, on days 1, 3, 10, and 21 post dry-off. Samples were centrifuged at 100,000 g for 60 min prepare ultra-whey's, depleted of immunoglobulins, digested Trypsin/Lys-C before to analysis by LC-MS/MS.
**Data source location**	National Animal Disease Center, USDA/ARS, Ames IA, 50,010 United States
**Data accessibility**	The mass spectrometry data and MaxQuant data have been deposited to the ProteomeXchange Consortium via the PRIDE. Repository name: ProteomeXchange Consortium via the PRIDE partner repository [2,3]. Data identification number: PXD017837. https://www.ebi.ac.uk/pride/archive/projects/PXD017837 Thermo raw file code for Pride raw files and supplemental Excel files. The 3 technical replicates are denoted as a letter A, B and C. The number following is the cow identification number for 11 cows used. The final two-digit number after the underscore is the day sampled where _01 = day 1, _03 = day 3, _10 = day 10 and _21 = day 21 of dry period. For example, A1313_01 is technical replicate A for cow 1313 collected on day 1. B1313_03 is technical replicate B for cow 1313 collected on day 3.
**Related research article**	Timothy A. Reinhardt* and John D. Lippolis, Characterization of Bovine Dry Secretions and their Proteome from the End of Lactation Through Day 21 of the Dry Period, Journal of Proteomics, in press.

**Value of the Data**

This dataset protein quantitative change in dry secretions as a cow's dry period and mammary involution proceeds through the first 21 days.
Dairy scientists with interest in mammary involution, and the interface of the dry period with the development of mastitis in the next lactation and accelerated involution approaches to reduce mastitis.
These data can be mined to help understand the factor responsible for slowed but variable bacterial growth in dry secretions and provide biomarkers for studying accelerated involution approaches to reduce mastitis.

## 1 Data description

All data have been deposited to the ProteomeXchange Consortium via the PRIDE partner repository with the dataset identifier PXD017837. Data output from MaxQuant presented there also. An Excel file provides untransformed data for each raw file after the MaxQuant analysis (see below).

This data is the basis of the publication “Characterization of bovine mammary gland dry secretions and their proteome from the end of lactation through day 21 of the dry period” [1].

## 2 Experimental design, materials, and methods

### 2.1 Animals and treatments

Eleven confirmed pregnant Holstein cows were used in the study. All animal protocols and procedures were approved by the Animal Care and Use Committee at the National Animal Disease Center, USDA/ARS using the National Institutes of Health guide for the care and use of Laboratory animals (NIH Publications No. 8023, revised 1978), Guide for Care and use of laboratory Animals (8th addition, 2011, and Guide for the care and use of Agricultural Animals in Teaching and Research (third edition 2010).

Complete details of the experiment, sample preparation, mass spectrometry procedures and data analysis can be found in the manuscript entitled “Characterization of bovine mammary gland dry secretions and their proteome from the end of lactation through day 21 of the dry period” [1].

Briefly, on day cows ceased to be milked, a maximum of 50 ml of dry secretion was collected aseptically from the same quarter on days 1, 3, 10 and 21 after cessation of milking. Samples were brought to the lab and centrifuged at 10,000 x g at 4 °C for 20 min to produce skim samples. This skim milk/dry secretion was centrifuged in a 50.2 Ti rotor 150,000 x g at 4 °C for 60 min to produce whey samples for proteomics.

Dry secretion whey samples were digested with Trypsin/Lys-C Mix, Mass Spec Grade (Promega Corporation, Madison, WI) using the two-step in-solution digestion protocol outline in manufacturer's instructions. The digests were concentrated and desalted using Pierce C18 spin columns (Thermo Fisher Scientific, West Palm Beach, FL)

The peptide digests were chromatographed on an Acclaim PepMap 100 C18, 3 μm, 75 μm x 50 cm column in mobile phase A (95% H2O: 5% acetonitrile and 0.1% formic acid) and mobile phase B (5% H2O: 95% acetonitrile and 0.1% formic acid) gradient, 1%B for 15 min, 1%−26% B from 15 to 195 min, 90% B from195 to 210 min, 90% B from to 220 min at 300nl/min, and back to 1% B from 220 to 275 at 500 nl/min. The analytical column was connected to a Nanospray Ion Source on the front end of an LTQ OrbiTrap Velos Pro (Thermo Fisher Scientific, West Palm Beach, FL) mass spectrophotometer. The capillary temperature was 275 °C and spray voltage optimized using the API stability evaluation software. Data-dependent method settings were; FTMS was 30,000 resolution from 300 to 2000 m/z followed by MSMS scans at 15,000 resolution, FTMS Top10. Activation was CID using a normalized collision energy of 35. The minimal signal required was 5000 and repeated mass exclusion duration of 60 s. All 11 cows and 4 time points per cow were in 3 technical replicates.

The raw data files from the mass spectrometer were inserted into MaxQuant (Version 1.6.7.0) [4]. The data was Normalization using the MaxQuant LFQ algorithm for label-free quantification [5]. The *Bos taurus* reference database was downloaded from Uniprot (March 2019). Data from MaxQuant was loaded into Perseus [6].

Significant GO, KEGG and Reactome terms were calculated within String v11 [7].

## Declaration of Competing Interest

The authors declare that they have no known competing financial interests or personal relationships which have, or could be perceived to have, influenced the work reported in this article.

## Appendix Supplementary materials

Image, application 1
